# Malaria prevalence, anemia and baseline intervention coverage prior to mass net distributions in Abia and Plateau States, Nigeria

**DOI:** 10.1186/1471-2334-14-168

**Published:** 2014-03-26

**Authors:** Gregory S Noland, Patricia M Graves, Adamu Sallau, Abel Eigege, Emmanuel Emukah, Amy E Patterson, Joseph Ajiji, Iheanyichi Okorofor, Oji Uka Oji, Mary Umar, Kal Alphonsus, James Damen, Jeremiah Ngondi, Masayo Ozaki, Elizabeth Cromwell, Josephine Obiezu, Solomon Eneiramo, Chinyere Okoro, Renn McClintic-Doyle, Olusola Oresanya, Emmanuel Miri, Paul M Emerson, Frank O Richards

**Affiliations:** 1 The Carter Center, 453 Freedom Parkway, Atlanta, GA 30307, USA; 2 The Carter Center, Jos, Plateau State, Nigeria; 3 The Carter Center, Southeast Owerri, Imo State, Nigeria; 4 Plateau State Ministry of Health, Jos, Nigeria; 5 Abia State Ministry of Health, Umuahia, Nigeria; 6 University of Jos, Jos, Nigeria; 7 Federal Medical Centre, Owerri, Imo State, Nigeria; 8 Federal Ministry of Health, Abuja, Nigeria; 9 Current address: School of Public Health, Tropical Medicine and Rehabilitation Sciences, James Cook University, Cairns, QLD, Australia; 10 Current address: Agnes Scott College, Decatur, GA, USA

**Keywords:** Malaria, Plasmodium, Falciparum, Malariae, Anemia, Net use, Net ownership, Nigeria, LLIN, Bed net

## Abstract

**Background:**

Nigeria suffers the world’s largest malaria burden, with approximately 51 million cases and 207,000 deaths annually. As part of the country’s aim to reduce by 50% malaria-related morbidity and mortality by 2013, it embarked on mass distribution of free long-lasting insecticidal nets (LLINs).

**Methods:**

Prior to net distribution campaigns in Abia and Plateau States, Nigeria, a modified malaria indicator survey was conducted in September 2010 to determine baseline state-level estimates of *Plasmodium* prevalence, childhood anemia, indoor residual spraying (IRS) coverage and bednet ownership and utilization.

**Results:**

Overall age-adjusted prevalence of *Plasmodium* infection by microscopy was similar between Abia (36.1%, 95% CI: 32.3%–40.1%; n = 2,936) and Plateau (36.6%, 95% CI: 31.3%–42.3%; n = 4,209), with prevalence highest among children 5-9 years. *P. malariae* accounted for 32.0% of infections in Abia, but only 1.4% of infections in Plateau. More than half of children ≤10 years were anemic, with anemia significantly higher in Abia (76.9%, 95% CI: 72.1%–81.0%) versus Plateau (57.1%, 95% CI: 50.6%–63.4%). Less than 1% of households in Abia (n = 1,305) or Plateau (n = 1,335) received IRS in the 12 months prior to survey. Household ownership of at least one bednet of any type was 10.1% (95% CI: 7.5%–13.4%) in Abia and 35.1% (95% CI: 29.2%-41.5%) in Plateau. Ownership of two or more bednets was 2.1% (95% CI: 1.2%–3.7%) in Abia and 14.5% (95% CI: 10.2%–20.3%) in Plateau. Overall reported net use the night before the survey among all individuals, children <5 years, and pregnant women was 3.4%, 6.0% and 5.7%, respectively in Abia and 14.7%, 19.1% and 21.0%, respectively in Plateau. Among households owning nets, 34.4% of children <5 years and 31.6% of pregnant women in Abia used a net, compared to 52.6% of children and 62.7% of pregnant women in Plateau.

**Conclusions:**

These results reveal high *Plasmodium* prevalence and childhood anemia in both states, low baseline coverage of IRS and LLINs, and sub-optimal net use—especially among age groups with highest observed malaria burden.

## Background

In Nigeria, approximately 97% of the estimated 160 million inhabitants are at risk of *Plasmodium* infection [1], resulting in an estimated 51 million cases and 207,000 deaths annually—more than any other country in the world and approximately 25% of the total malaria burden within Africa [2]. Malaria reportedly accounts for an estimated 60% of outpatient visits in Nigeria, 30% of hospitalizations, 30% of under-five mortalities, 25% of infant mortalities and 11% of maternal mortalities [3]. Beyond the impact on human health, malaria exerts a large economic burden on individuals and households, with the loss due to protection, treatment and indirect costs estimated to consume an estimated 132 billion Naira ($835 million) [4].

In 2008, the Nigerian Ministry of Health committed to an ambitious goal of reducing by 50% malaria-related morbidity and mortality by 2013 [5]. This is to be achieved through scale-up for impact (SUFI) of World Health Organization (WHO)-recommended prevention and control measures in order to provide protection for all at-risk Nigerians. Specific targets set by the National Malaria Control Strategic Plan for 2009-2013 include: at least 80% of households own two or more insecticide treated nets (ITN) by 2010; at least 80% of pregnant women and children under five years of age sleep under an ITN nightly by 2010; at least 8% (by 2010) and 20% (by 2013) of households in targeted areas receive indoor residual spraying (IRS); and 100% of pregnant women attending antenatal care clinics receive two doses of intermittent preventative therapy (IPTp) by 2013.

Use of ITNs is considered one of the most cost-effective interventions against malaria in highly endemic areas [6]. ITNs are associated with significant reductions in malaria morbidity and mortality [7], reduced complications associated with malaria in pregnancy [8], and reduced all cause mortality [7]. In 2008, 8.0% of households in Nigeria owned at least one ITN and only 2.7% owned two or more ITNs, while use of ITNs was 6% among children under five years and 5% among pregnant women [3]. Beginning in 2009, the National Malaria Control Programme launched a two-pronged strategy for distribution of long lasting insecticidal nets (LLINs) across the country’s 36 states and Federal Capital Territory (FCT). The first ‘catch-up’ phase aimed to rapidly scale up LLIN ownership through mass campaigns targeting the distribution of 64 million LLINs (2 nets for each of 32 million households). The second ‘keep-up’ phase involves the expansion of routine distribution channels in order to sustain the high-level coverage attained through universal mass distribution. Routine channels, which include antenatal care clinics, immunization clinics, school-based distributions, and the commercial sector, had been utilized under previous national strategic plans to provide nets to pregnant women and children under five years—populations considered most vulnerable to malaria.

**Figure 1:**
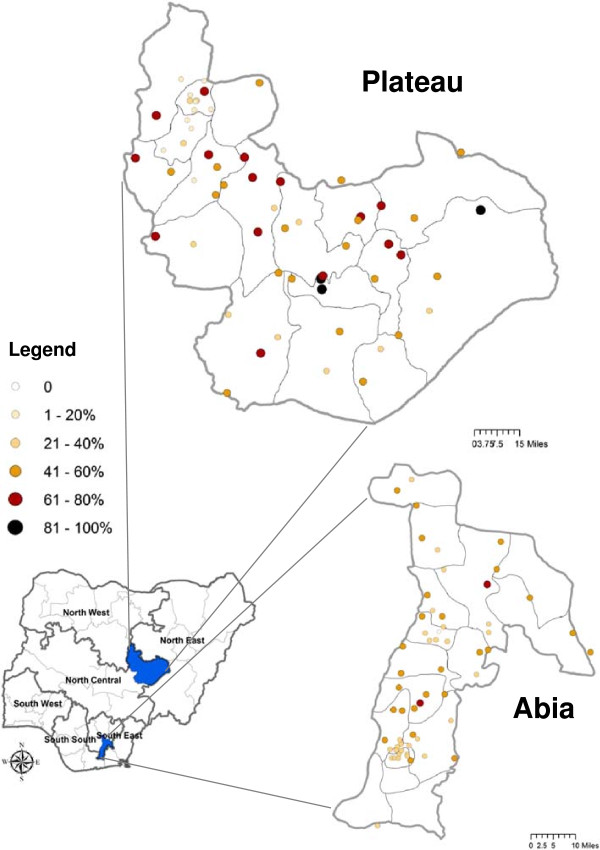
**Study areas and *Plasmodium *prevalence by cluster. ***Plasmodium* prevalence, diagnosed by microscopy, by survey cluster site in Abia and Plateau States, Nigeria. State maps outline their constituent local government areas (LGAs), while the national map highlights the six geo-political zones within Nigeria.

Administratively, Nigeria’s 36 states are divided into six geo-political zones (Figure 1). The national demographic and health surveys (DHS) of 2003 [9] and 2008 [3] provided net coverage estimates at the national and geo-political zone level, while the national malaria indicator survey (MIS) of 2010 [1] provided national and zonal estimates of malaria intervention coverage as well as parasite prevalence in children under five years. However, surveys designed to evaluate the scale-up of malaria interventions and parasite prevalence amongst all age groups with state-level precision are lacking.

The Carter Center has helped to support net distribution efforts in Nigeria since 2004. This began with integrated ITN distribution during mass drug administration (MDA) for lymphatic filariasis and onchocerciasis [10]. Since anopheline mosquitoes can transmit both *Plasmodium* and *Wuchereria bancrofti* parasites, nets provide protection and reduce transmission of both diseases simultaneously, while enabling programmatic efficiency and cost savings [11,12]. In order to help evaluate the impact of mass LLIN distribution in Abia and Plateau States, The Carter Center coordinated a modified malaria indicator survey on behalf of the state ministries of health in September 2010, prior to the states’ planned mass distribution campaigns. The goal of this survey was to determine baseline state-level estimates of net ownership and utilization, *Plasmodium* prevalence in all age groups and anemia prevalence in children less than 11 years old in Abia and Plateau States, Nigeria.

## Methods

### Study area and sample selection

This study was conducted in September 2010 in Abia State (population est. 3.2 million) located in the South East Zone and Plateau State (population est. 3.6 million) located in North Central Zone (Figure 1). Malaria transmission in Abia is predicted to typically last ten months or longer (March-December), while in Plateau, a shorter seven-month seasonal transmission period predominates (May-November) [13].

A random cluster sampling design was used to select a state-level representative sample in each state. The required sample size was based on detection of a 50% prevalence of malaria in children under five years with 5% precision, α = 0.05 and a design effect of 2. Assuming a 70% response rate and that 80% of households include at least one child less than five years of age, approximately 1400 households were required per state. Clusters were defined as a census enumeration area (EA), or a randomly selected segment of large EAs, with an expected average of 25 households per cluster. Using a list of all EAs within each state obtained from the Nigerian National Population Commission, 60 clusters per state were selected in systematic (equal interval) fashion with a random start. Survey teams made a rough listing and sketch map of household locations within each cluster, and if the number of households exceeded pre-defined thresholds, the cluster was randomly divided into segments and one segment randomly selected according to the Multiple Indicator Cluster Survey (MICS) methods [14]. All households within selected clusters were eligible for inclusion in the study. If no one was home at the time of first visit, interviewers returned later in the day in an attempt to include all eligible households.

A household was defined as: a married man, his wives and all of his dependents who currently live with him (including biological children, adoptive children, domestic workers, other family members for whom he is responsible); an unmarried (widowed, divorced, never married) woman who is recognized as the head of household and all of her dependents who currently live with her; or two or more unmarried adult persons who sleep in the same dwelling unit and who share meals (e.g. university students who share an apartment).

### Survey questionnaire

The survey questionnaire was based on the Roll Back Malaria Monitoring & Evaluation Reference Group (MERG) Malaria Indicator Survey household and women’s questionnaires, modified for local conditions [15]. The questionnaires were translated and printed in Hausa and Igbo languages, and field tested prior to the survey. Household interviews were conducted with consenting heads of households or another resident adult if the head of household was absent or unable to respond. Respondents were asked about demographic information of usual residents, standard socio-economic indicators, educational level, household construction, indoor residual spraying as well as mosquito net ownership, utilization, condition and care (verified by direct observation). One woman of reproductive age (15–49 years) from each household was selected at random to answer the women’s questionnaire, which included questions relating to malaria knowledge, attitudes, practice, and exposure to malaria health messages. Geo-coordinates of each household were recorded using handheld global positioning system units (Garmin eTrex H, Garmin International).

### Blood testing

All children ten years of age or younger as well as individuals of all ages in every third household were eligible for malaria parasite testing by rapid diagnostic test (RDT) and microscopy. RDT testing from finger prick samples was used for on-site diagnosis and treatment of malaria. CareStart Malaria HRP2/pLDH combo RDTs (Access Bio, Inc., model G0131), which can discriminate non-*P. falciparum* infections from pan-*Plasmodium* infections, were used according to the manufacturer’s instructions. Thick and thin blood films were prepared by laboratory technologists on a single slide, air dried and stained with Giemsa on the day of collection. Blood films were read by certified laboratory scientists in The Carter Center laboratory in Owerri, Imo State (for Abia samples) and The Carter Center laboratory in Jos, Plateau State (for Plateau samples). A WHO-certified microscopist then re-read all positive slides and 10% of the negatives from both states for quality control. Individuals with positive RDT results were offered on-site treatment according to national guidelines: artesunate-amodiaquine (Sanofi-Aventis Groupe) or artemether-lumefantrine (Coartem, Novartis AG) for non-pregnant individuals older than four months of age, or sulfadoxine-pyrimethamine for self-reported pregnant women. Individuals younger than four months with a positive RDT test were referred to the nearest health facility for further evaluation, as were RDT-negative individuals with self-reported fever or other overt signs of clinical illness. To enable maximum participation for blood sampling, households with absentees were revisited later the same day to recruit individuals missing at the first visit.

Blood samples were also used for anemia testing in all children under 11 years of age using handheld spectrophotometers (Hb201+, HemoCue, Inc.). Anemia was classified according to WHO guidelines [16] using altitude-adjusted hemoglobin (Hb) values: mild (10.0 g/dL ≤ Hb < 11.0 g/dL), moderate (7.0 g/dL ≤ Hb < 10.0 g/dL), severe (Hb <7.0 g/dL) for children less than five years; and mild (11.0 g/dL ≤ Hb < 11.5 g/dL), moderate (8.0 g/dL ≤ Hb < 11.0 g/dL), severe (Hb <8.0 g/dL) for children 5–11 years. Individuals with moderate anemia were provided treatment according to national guidelines: presumptive anti-malarial chemotherapy (artesunate-amodiaquine or artemether-lumefantrine), iron supplementation (iron syrup for those between four months and five years or iron-folate tablets for children less than five years), and a single dose 400 mg albendazole for children less than two years. Individuals with severe anemia were referred directly to the nearest health facility for evaluation and treatment.

### Data analysis

Completed questionnaires were checked by supervisors in the field and inconsistencies verified with the respondents. Data were double entered by different clerks in each state and compared for consistency using EpiInfo™ v3.5.3 (Centers for Disease Control and Prevention). Statistical analysis was conducted using Stata v11.2 (StataCorp LP). Point estimates and confidence intervals were derived using the SURVEY (SVY) commands in Stata to account for clustering and sampling weights. Logistic regression models under the SVY command were used to calculate *Plasmodium* prevalence estimates for each state adjusted for age, clustering and sampling weights.

A household wealth index was constructed using the methods of Vyas and Kumaranayake [17]. This index was based on possession of assets (having electricity in the household, a functioning radio and/or a functioning television), type and location of usual water source, possession of and type of latrine, house construction materials (wall, roof and floor), number of rooms and density of people per room. The first principal component was used to generate the asset index, which was then divided into quintiles. The indicators “percent of household with at least one net for every two people” and “percent of population with access to a net within a household” were calculated using the methods of Kilian and colleagues [18].

### Ethics considerations

This protocol received ethical clearance from the Emory University Institutional Review Board (IRB#00044684), and the Nigeria Health Research Ethics Committee [NHREC/01/01/2007]. Verbal informed consent to participate in the household and women’s interviews was sought from heads of household or eligible women, respectively. For blood testing, verbal informed consent was sought from all eligible individuals older than 18 years of age or from the parents of minors (0–17 years of age), as well as additional verbal assent from minors over the age of six years.

## Results

### Characteristics of study population

A total of 116 out of the selected 120 clusters were sampled in Abia and Plateau States (Figure 1). Two clusters each in Abia and in Plateau were not accessible due to civil unrest or insecurity. Missing clusters were not replaced. Sampled clusters contained 1,426 households in Abia (mean number of households per cluster: 24.6; range: 9–40) and 1,382 households in Plateau (mean number of households per cluster: 23.8; range: 7–38). Of eligible households, 121 (8.5%) in Abia and 47 (3.4%) in Plateau were excluded from final analysis because no one was present or because of refusal. This resulted in a study population of 1,305 households in Abia and 1,335 in Plateau.

Characteristics of surveyed households and individuals are shown in Table 1. The mean number of individuals per household and number of rooms used for sleeping were lower in Abia than in Plateau. The mean elevation of surveyed households was also significantly lower in Abia (108.0 m; range: 11 m–351 m) versus Plateau (815.0 m; range: 117 m–1344 m). Households in Abia were most commonly (34.3%) classified in the highest wealth index category, while households in Plateau were most commonly (28.0%) classified in the lowest wealth classification.

**Table 1:** Characteristics of study households and individuals in Abia and Plateau states, Nigeria, September 2010

	**Abia**	**Plateau**
Number of clusters sampled	58	58
***Household characteristics***		
Number of households sampled	1,305	1,335
Mean (SD) number of people per household	4.4 (2.7)	6.3 (3.1)*
Mean (SD) number of sleeping rooms per household	1.9 (1.1)	2.4 (1.2)*
Altitude		
≤100 m (%)	55.1	0.0*
100–1000 m (%)	44.8	46.3
>1000 m (%)	0.2	53.7*
Household wealth index, quintiles		
Poorest (%)	6.9	28.0*
Second (%)	11.8	25.7*
Third (%)	21.2	17.0
Fourth (%)	25.9	18.0
Richest (%)	34.3	11.3*
***Individual characteristics***		
Number of persons in sampled households	5,754	8,303
Age		
<5 yrs (%)	14.7	16.4
5-9 yrs (%)	12.0	16.0*
10-14 yrs (%)	9.9	11.9
15-19 yrs (%)	9.3	9.8
20-49 yrs (%)	35.5	36.7
≥50 yrs (%)	18.6	9.3*
Proportion female (%)	53.6	49.6
Pregnant women, self-reported (% of all individuals/% of women)	2.5/4.6	2.1/4.3

*Asterisk indicates statistically significant difference between states (non-overlapping 95% confidence intervals).

Most houses in Abia were made of cement or stone block (81.4%), with a minor proportion built of mud and sticks (14.2%) or mud bricks (2.8%), whereas in Plateau, a greater diversity of construction types was observed between cement or stone blocks (40.9%), mud bricks (33.7%), mud and stick construction (16.7%), and solid brick construction (8.6%). The majority of roofs in both Abia and Plateau were made of zinc or metal (87.4%, 70.9%, respectively) with the remained thatch/palm leaf roofs (9.1%, 27.5%, respectively) or concrete/cement roofs (1.0%, 1.1%, respectively).

Demographic data for the 14,057 individuals living in the study households is also shown in Table 1. Age distribution of individuals in surveyed households was generally similar between states, as were the proportions of males, females and self-reported pregnant women. There were significantly more females (53.6%) than males (49.6%) in Abia, but not in Plateau (49.5% and 50.5%, respectively).

### *Plasmodium* prevalence

All children less than eleven years of age and individuals of all ages in every third household were eligible for malaria parasite testing by microscopy and rapid diagnostic test (RDT). Microscopy results were available from 2,936 individuals in Abia and 4,209 individuals in Plateau. Overall age-adjusted prevalence of *Plasmodium* as detected by microscopy was similar between Abia (36.1%, 95% CI: 32.3%–40.1%) and Plateau (36.6%, 95% CI: 31.3%–42.3%). Crude prevalence by cluster ranged from 14.8% to 66.1% in Abia and from 1.8% to 86.4% in Plateau (Figure 1). Cluster prevalence was negatively, but poorly, associated with elevation in Abia (*r*^2^ = 0.014) and Plateau (*r*^2^ = 0.142).

**Figure 2:**
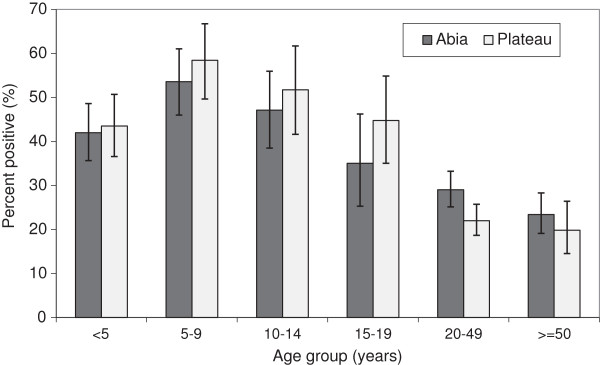
***Plasmodium *prevalence.** Proportion of individuals testing positive for *Plasmodium* infection by microscopy, by age group, Abia and Plateau States, Nigeria, September 2010. In Abia, 68.1% of all infections were *P. falciparum*, 32.0% *P. malariae* with one co-infection; in Plateau, 98.7% of infections were *P. falciparum*, 1.4% *P. malariae* with two co-infections. Error bars are 95% confidence intervals.

In Abia, 68.1% of infections were identified as *Plasmodium falciparum*, 32.0% were *Plasmodium malariae* with one instance of *P. falciparum*-*P. malariae* co-infection. In Plateau, 98.7% of infections were *P. falciparum*, 1.4% *P. malariae* and two instances of co-infection. No *Plasmodium ovale* infections were identified in either state. Prevalence of *Plasmodium* infection was significantly associated with age in both Abia (*χ*^2^ = 136.62, P < 0.001) and Plateau (*χ*^2^ = 326.45, P < 0.001), with prevalence highest in the 5–9 year age group and lowest in those aged 50 years and older (Figure 2). Infection was non-significantly higher in males in both states, and significantly and inversely associated with wealth in Abia (*χ*^2^ = 122.96, P < 0.001) and Plateau (*χ*^2^ = 318.25, P < 0.001).

Blood samples were tested by RDT for on-site provision of treatment for positive individuals. Concordant results were obtained from 6,502 of 6,771 samples with valid results for both microscopy and RDT (96.0% agreement; κ = 0.919). Overall age-adjusted prevalence of *Plasmodium* as determined by RDT was similar between Abia (30.4%, 95% CI: 25.7%–35.4%) and Plateau (32.4%, 95% CI: 26.6%–38.9%), with a significantly higher proportion of non-*P. falciparum* infections in Abia (40.0%) than in Plateau (0.7%), in line with microscopy results.

### Anemia

Mean unadjusted hemoglobin among children less than 11 years of age was significantly lower in Abia (9.9 g/dL, 95% CI: 9.7–10.1 g/dL) versus Plateau (10.9 g/dL, 95% CI: 10.6–11.2 g/dL). After adjusting for age and altitude, more than half of children were anemic (any type) in both states (Table 2), with anemia significantly more prevalent in Abia (76.9%, 95% CI: 72.1%–81.0%) than in Plateau (57.1%, 95% CI: 50.6%–63.4%). In Abia, there was a significantly greater proportion of children with both moderate anemia and severe anemia compared to Plateau. Anemia was significantly more prevalent among children less than 5 years (64.7%, 95% CI: 58.3%–70.6%) compared to children five years and older (50.3%, 95% CI: 43.0%–57.6%) in Plateau, with a similar trend observed in Abia (80.5%, 95% CI: 75.1%–84.9%; 73.1%, 95% CI: 67.4%–78.2%).

**Table 2:** Anemia prevalence in children less than 11 years of age in Abia and Plateau states, Nigeria, September 2010

	**Abia (n = 1,556)**	**Plateau (n = 2,823)**
	**% (95% CI)**	**% (95% CI)**
Normal^1^	23.2 (19.0–27.9)	45.0 (38.2–52.0)*
Mild^2^	16.1 (14.0–18.3)	18.5 (16.7–20.4)
Moderate^3^	53.1 (47.8–58.3)	33.6 (28.1–39.5)*
Severe^4^	7.8 (5.8–10.3)	3.0 (2.3–3.8)*

^1^For age <5 years: hemoglobin [Hb] ≥ 11.0 g/dL; for age 5–11 years: Hb ≥ 11.5 g/dL.

^2^For age <5 years: 10.0 g/dL < Hb < 11.0 g/dL; for age 5–11: 11.0 g/dL < Hb < 11.5 g/dL.

^3^For age <5 years: 7.0 g/dL < Hb < 10.0 g/dL; for age 5–11: 8.0 g/dL < Hb < 11.0 g/dL.

^4^For age <5 years: Hb <7.0 g/dL; for age 5–11: Hb <8.0 g/dL.

*Asterisk indicates statistically significant difference between states (non-overlapping 95% confidence intervals).

### Malaria prevention measures

Less than one percent of households in Abia (0.4%) and Plateau (0.6%) reported that indoor residual spraying (IRS) with insecticide had been performed in the past 12 months (Table 3).

**Table 3:** Household malaria prevention measures, Abia and Plateau states, Nigeria, September 2010

	**Abia (n = 1,305)**	**Plateau (n = 1,335)**
Percent of households that received IRS in past 12 months (95% CI)	0.4% (0.1–1.4)	0.6% (0.3–1.5)
Percent of households owning at least one net (95% CI)	10.1% (7.5–13.4)	35.1% (29.2–41.5)*
Percent of households owning two or more nets (95% CI)	2.1% (1.2–3.7)	14.5% (10.2–20.3)*
Percent of households with at least one net for every two people (95% CI) – all households	1.4% (0.8–2.1)	6.3% (3.8–8.8)*
Percent of households with at least one net for every two people (95% CI) – households with at least one net	14.2% (8.8–19.6)	18.1% (13.3–23.0)
Mean number of nets per household (95% CI) – all households	0.1 (0.1–0.2)	0.6 (0.4–0.7)*
Mean number of nets per household (95% CI) – households with at least one net	1.2 (1.1–1.3)	1.7 (1.5–1.8)*
Percent of population with access to net within a household (assuming net used by two people) (95% CI)	8.2% (3.7-6.9)	23.8% (14.5-24.1)*

*Asterisk indicates statistically significant difference between states (non-overlapping 95% confidence intervals).

Household ownership of at least one bednet (of any type) was 10.1% in Abia and 35.1% in Plateau. Only 2.1% of households in Abia and 14.5% of households in Plateau owned two or more nets. Likewise, 1.4% and 6.3% of all households in Abia and Plateau, respectively, owned at least one net for every two household members and 14.2% and 18.1% of households that owned nets in Abia and Plateau, respectively, had enough nets for every two persons. While net ownership was greatest among the highest wealth index category in both states, there was not a significant association between net ownership and quintiles of household wealth index in either Abia (*χ*^2^ = 9.01, P = 0.28) or Plateau (*χ*^2^ = 17.29, P = 0.17).

The mean number of nets per household was significantly lower in Abia versus Plateau, whether considering all households (0.1 nets versus 0.6 nets) or only those households that owned at least one net (1.2 nets versus 1.7 nets), Table 3. The most frequently reported reasons for not owning a net in households without nets include: nets not available (56.3% in Abia, 44.3% in Plateau), nets are too expensive (25.4%; 35.9%). Very few people reported not liking nets (0.9% in Abia, 1.7% in Plateau) or no mosquitoes (5.2%, 1.7%) as reasons for not owning nets. Overall, 8.2% of the population in Abia and 23.8% of the population in Plateau had access to a net within a household, assuming a net is used by two people (Table 3).

**Figure 3:**
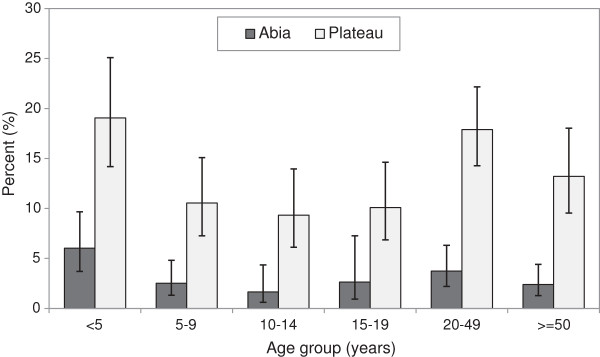
**Net use.** Proportion of individuals who reported sleeping under a net the night prior to survey, by age group, Abia and Plateau States, Nigeria, September 2010. Error bars are 95% confidence intervals.

As shown in Table 4, reported net use the night prior to the survey among all individuals in all households was significantly lower in Abia (3.4%, 95% CI: 2.1% – 5.5%) versus Plateau (14.7%, 95% CI: 11.3% – 18.9%). Reported net use was positively associated with wealth in Plateau (*χ*^2^ = 172.67, P = 0.002), but not Abia (*χ*^2^ = 40.64, P = 0.17). Reported net use was also significantly associated with age in both Abia (*χ*^2^ = 29.93, P = 0.007) and Plateau (*χ*^2^ = 101.22, P < 0.001), with net use highest among children less than five years in both states (Figure 3). Reported net use was significantly lower in Abia for all age groups except those 15–19 years. Reported net use among pregnant women was also lower in Abia (5.7%) versus Plateau (21.0%). There was no difference in net use between males and females in either Abia (*χ*^2^ = 1.35, P = 0.30) or Plateau (*χ*^2^ = 1.92, P = 0.12).

**Table 4:** Reported net use, Abia and Plateau states, Nigeria, September 2010

	**Abia**		**Plateau**	
	***n***	**% (95% CI)**	***n***	**% (95% CI)**
***All households***				
All individuals	5,754	3.4 (2.1–5.5)	8,303	14.7 (11.3–18.9)*
Children <5 years	853	6.0 (3.7–9.6)	1,384	19.1 (14.2–25.0)*
Pregnant women (self-reported)	129	5.7 (1.9–15.8)	186	21.0 (14.3–29.8)
***Households owning at least 1 net***				
All individuals	648	27.6 (20.1–36.6)	3,161	41.1 (36.5–45.9)
Children <5 years	135	34.4 (24.2–46.2)	534	52.6 (44.2–60.9)
Pregnant women (self-reported)	19	31.6 (12.0–61.0)	72	62.7 (49.2–74.4)

*Asterisk indicates statistically significant difference between states (non-overlapping 95% confidence intervals).

The same trends were observed when restricted only to households owning at least one net. Net use was lower in Abia versus Plateau among all individuals (27.6%, 95% CI: 20.1%–36.6%; vs. 41.1%, 95% CI: 36.5%–45.9%, respectively), children under five years (34.4% vs. 52.6%) and pregnant women (31.6% vs. 62.7%) (Table 4). In this smaller subset, associations between net use and age (*χ*^2^ = 11.25, P = 0.14), gender (*χ*^2^ = 1.30, P = 0.26) and wealth (*χ*^2^ = 10.80, P = 0.50) were not evident in Abia. In Plateau, significant associations with age (*χ*^2^ = 143.79, P < 0.001) and wealth (*χ*^2^ = 67.09, P = 0.009), but not gender (*χ*^2^ = 3.17, P = 0.10) were observed.

Characteristics of nets observed in Abia (n = 150) and Plateau (n = 807) are summarized in Table 5 along with confidence intervals for all point estimates. The majority of nets in both states were identified as LLINs, with a significantly lower proportion in Abia (65.6%) versus Plateau (89.5%). Baby nets (nets on a folding free standing wire frame, suitable for placing over a sleeping infant) comprised a significantly greater proportion of all nets in Abia (17.4%) compared to Plateau (0.5%). Approximately half of all nets in Abia (49.4%) and Plateau (50.7%) were reportedly obtained less than 12 months prior to the survey. In Abia, nets were most frequently obtained from health facilities (37.0%) and markets (32.2%). Other sources include shops (14.4%), received as a gift (8.7%), mass distribution campaigns (4.4%), and community health worker apart from mass distribution (2.2%). In Plateau, nets were also most frequently obtained from markets (38.4%) and health facilities (30.3%). The remainder were obtained from mass distribution campaigns (17.4%), received as a gift (6.5%), shops (2.4%), various other source (2.4%) and community health worker (1.9%). Around half of all nets in both Abia (49.0%) and Plateau (47.5%) were reportedly purchased.

**Table 5:** Net characteristics in sampled households, Abia and Plateau states, Nigeria, September 2010

	**Abia (n = 150)**	**Plateau (n = 807)**
	**% (95% CI)**	**% (95% CI)**
Net type		
LLINs	65.6 (54.0–75.5)	89.5 (84.2–93.1)*
Pre-treated net	0.8 (0.1–5.8)	0.4 (0.1–1.4)
Untreated net	5.8 (1.1–25.0)	3.3 (1.9–5.7)
Other	0	0.3 (0.1–1.0)*
Don’t know or missing	27.8 (19.3–38.3)	6.5 (3.6–11.6)*
Proportion baby nets	17.4 (9.3–30.3)	0.5 (0.2–1.5)*
Net age		
≤ 6 months	26.6 (18.5–36.5)	28.5 (20.2–38.6)
6-12 months	22.8 (13.7–35.4)	22.2 (16.9–28.5)
> 12 months	43.2 (32.8–54.1)	47.2 (39.6–55.0)
Don’t know or missing	7.5 (3.9–13.9)	2.1 (1.2–3.6)*
Source of nets		
Market	32.2 (20.5–46.4)	38.4 (28.7–49.3)
Health facility	37.0 (24.2–51.9)	30.3 (22.1–40.1)
Mass distribution	4.4 (1.4–12.8)	17.4 (9.0–30.9)
Shop	14.4 (4.7–36.5)	2.4 (1.1–5.4)
Gift	8.7 (4.6–15.9)	6.5 (3.9–10.6)
Community health worker (non-campaign)	2.2 (0.7–6.2)	1.9 (1.0–3.8)
Other	0.9 (0.2–3.6)	2.4 (1.0–6.0)
Proportion of nets purchased	49.0 (37.5–60.6)	47.5 (37.6–57.7)
Proportion of nets ever used	74.7 (64.5–82.7)	86.1 (80.0–90.5)
Proportion nets observed hanging	52.3 (40.9–63.5)	79.2 (72.5–84.7)*
Proportion of hanging nets hung at appropriate height	95.2 (82.0–98.9)	97.7 (94.5–99.0)
Reasons why net was not hung (multiple responses possible)		
Do not want to use net	40.0 (22.5–60.5)	28.4 (16.2–44.8)
Have not yet permanently hung	14.6 (7.5–26.5)	12.9 (5.0–29.5)
Too tired to hang	9.1 (3.2–23.1)	5.6 (1.6–18.2)
Don’t know how to hang	10.9 (4.4–24.5)	2.2 (0.8–6.0)
Inconvenient	3.6 (1.0–12.6)	7.3 (3.4–14.8)
Too hard to hang	7.3 (1.8–25.6)	3.3 (1.2–9.2)
No space for net	5.5 (0.9–27.8)	2.8 (0.8–9.2)
Person responsible for hanging absent	1.8 (0.2–12.4)	0.6 (0.1–4.3)
Other	20.0 (8.8–39.3)	31.4 (21.4–43.6)
Proportion nets used last night	60.6 (49.9–70.4)	80.4 (73.2–86.0)*
Reasons why net was not used last night (multiple responses possible)		
Usual user did not sleep here last night	17.4 (8.2–33.3)	1.6 (0.5–5.3)*
Net not needed last night	4.4 (1.2–14.5)	14.1 (9.1–21.2)
Cannot hang net	12.0 (6.1–22.3)	6.4 (3.0–13.1)
No mosquitoes	8.7 (3.9–18.4)	9.4 (3.4–23.7)
Net too old or torn	13.0 (6.2–25.5)	4.7 (1.9–10.9)
Net not available last night (washing)	3.3 (0.4–21.2)	12.5 (6.2–23.8)
Too hot	10.9 (3.9–26.7)	3.3 (1.2–9.1)
Feel closed in or afraid	6.5 (1.8–21.3)	5.3 (1.2–19.9)
No malaria	0	5.7 (1.5–19.6)*
Net too dirty	4.4 (1.4–13.1)	0.6 (0.1–3.6)
Don’t like smell	2.2 (0.3–14.5)	2.6 (0.9–7.5)
Other	20.7 (11.4–34.5)	24.7 (14.8–38.3)
Don’t know	4.4 (0.9–19.4)	7.9 (2.8–20.4)
Proportion nets with holes	17.7 (10.1–29.2)	16.5 (12.0–22.2)
Proportion nets with mends	10.7 (4.1–24.8)	5.4 (3.5–8.4)

*Asterisk indicates statistically significant difference between states (non-overlapping 95% confidence intervals).

The majority of nets in Abia (74.7%) and Plateau (86.1%) were reportedly used at least once. The proportion of nets hanging at the time of survey was significantly lower in Abia (52.3%) compared to Plateau (79.2%). Nearly all of the hanging nets in both states (95.2%; 97.7%, respectively) were hung at an appropriate height--i.e. able to be tucked in under sleeping mat on floor or mattress on bed. The most commonly reported reasons for not hanging nets in each state included: respondent did not want to use net (40.0% in Abia, 28.4% in Plateau), have not yet hung it (14.6%; 12.9%), were too tired to hang it last night (9.1%; 5.6%) and don’t know how to hang it (10.9%; 2.2%). Various other reasons were reported by less than 10% of respondents as shown in Table 5. The majority of nets were reportedly used by a household member the previous night in Abia (60.6%) and Plateau (80.4%), with use significantly higher in Plateau. Reasons why nets were not used last night are listed in Table 5. No single reason was reported by more than 18% of respondents. The proportions of nets with holes were similar in Abia (17.7%) and Plateau (16.5%), as were the proportions of nets with mends (10.7%; 5.4%, respectively).

Additional reported methods used for protection against mosquitoes or other nuisance insects include: mosquito coils (used by 37.7% and 27.8% of households in Abia and Plateau, respectively), canned insect spray (31.4%; 22.9%), “Otapiapia”, an organophosphate-based pesticide in liquid form (dichlorvos), approved for use in grain storage areas but widely available locally from shops and traders (19.5%; 38.6%; significantly higher in Plateau), “Piff Puff”, a synthetic pyrethroid-containing insecticide powder (9.9%; 12.8%). Other reported methods such as burning leaves (<6% of households) and using repellent soaps or creams (<1%) were uncommon in either state.

## Discussion

This study was conducted in September 2010 prior to planned mass distribution campaigns in Abia and Plateau States, which took place in August 2012 and December 2010, respectively. The results document high levels of *Plasmodium* infection and anemia in both states, extremely low (<1%) IRS coverage and low bed net ownership and use. Low IRS coverage across the sampled population is not unexpected, as IRS in Nigeria is limited to ‘target’ areas including: densely populated municipalities, areas with short malaria transmission seasons, areas where LLINs are difficult to implement, and institutional locations [5].

Around the same time (October 2010), the first national malaria indicator survey (MIS) was conducted throughout Nigeria in order to evaluate the scale-up of malaria prevention and control measures [1]. While the MIS provides national and zone-level estimates for interventions, state-level evaluation is also critical as mass net distribution campaigns are done on a state-by-state basis. In addition, since state campaigns have been conducted in different years, many of the 2010 MIS aggregate zonal estimates combine data from states that had already completed mass LLIN distribution with others that had not. As far as we are aware, this study is the first to report baseline estimates of malaria prevention measures, malaria prevalence and anemia in individual Nigerian states prior to scaled–up mass distribution campaigns targeting universal coverage.

Overall age-adjusted *Plasmodium* prevalence by microscopy (all ages) was similar between Abia (36.1%) and Plateau (36.6%) with almost one third of infections in Abia state being *P.malariae.* These represent some of the only modern estimates of *Plasmodium* prevalence across all age groups in Nigeria, as recent surveys including 2010 MIS tend to focus on specific sub-populations like children [1,19,20], neonates [21,22], pregnant women [23], or those infected with HIV [24-26]. Prevalence estimates for children under five years by microscopy were similar for Plateau (43.5%, 95% CI: 36.6%–50.7%) compared to the 2010 MIS estimate for the larger area of the North Central zone in which it is located (49.4%, CI not reported), but likely different in Abia (42.0%, 95% CI: 35.7%–48.6%) compared to the 2010 MIS South East zone estimate (27.6%, CI not reported) [1]. One recent study conducted among all aged individuals during the dry season in Lagos State, South West zone, estimated an overall prevalence of 14.7%, with prevalence highest in the 5–14 year age group [27]. This trend with age is consistent with our results, where prevalence was highest in the 5–9 and 10–14 age groups in both Abia and Plateau.

A high level of concordance was observed in *Plasmodium* prevalence between microscopy and RDT, though RDT-estimates were slightly lower than microscopy. This contrasts with observations from other large surveys that consistently observe higher RDT-prevalence attributed to antigen persistence following treatment or submicroscopic infections [28]. Differences between this study and MIS 2010 results in RDT-prevalence estimates for children under five years were less pronounced than for microscopy results. However, unlike our study, which utilized a Pf/Pan combination RDT, the MIS 2010 utilized Paracheck PF®, an RDT that only detects *P. falciparum*-specific histidine-rich protein-2. MIS RDT results thus likely underestimated the overall *Plasmodium* prevalence in some areas through undiagnosed non-falciparum infections, as nearly one third of malaria infections in Abia in the present study were identified as *P. malariae* by microscopy. A significant proportion of non-falciparum infections were also identified by microscopy in South East and North Central zones in the MIS 2010 [1]. Historically, a significant proportion of *P. malariae* and *P. ovale* infections were also reported by The Garki Project, in Kano State, North West zone, from 1969 to 1976 [29]. Taken together, these results demonstrate that non-falciparum infections are prevalent in parts of Nigeria and highlight the importance of utilizing multi-species RDTs to monitor trends of all *Plasmodium* parasites. In addition to variation in prevalence between species, our study highlights large heterogeneity in prevalence between clusters within states that deserves further investigation to improve malaria risk stratification of all species in Nigeria.

In this study, more than half of children less than 11 years in both states were found to be anemic (mild, moderate or severe), with prevalence higher in Abia than in Plateau and also higher among children less than 5 years. Our results are consistent with WHO’s estimate that two-thirds of preschool-age children in Africa are anemic [30], and within Nigeria, are similar to those from the 2010 MIS, which found 71.7% anemia prevalence in South East zone and 56.0% in North Central zone among children under five [1]. Malaria is a major cause of childhood anemia in malaria endemic areas where it accounts for approximately half of pediatric admissions for severe anemia [31,32]. Given the similar malaria prevalence between the two states, it is not immediately clear why anemia was significantly higher in Abia. Perhaps the higher prevalence of *P. malariae* or the slightly longer malaria transmission season may contribute. Other causes of anemia include iron and other nutritional deficiencies, blood disorders, inflammation and other acute and chronic diseases [16]. Thus differences in diet and genetic composition may also contribute to higher anemia in Abia. However, we hypothesize that repeated statewide MDA in Plateau, but not Abia, of deworming drugs from 2003–2012 for the elimination of lymphatic filariasis, as well as since 2008 for treatment of schistosomiasis in school-age children [33,34], may have reduced the prevalence of helminth infections that have been shown to interact with malaria infection to worsen anemia [35]. Indeed, a recent survey of school-aged children has confirmed a higher prevalence of hookworm infection in Abia compared to Plateau (D. Evans, personal communication).

Prior to 2009, Nigeria’s policy was to provide free net distribution to children under five and pregnant women (vulnerable groups) only. As part of Nigeria’s aim to reduce by 50% malaria-related morbidity and mortality by 2013, the country embarked in 2009 on a strategy of scaled-up mass distribution of universal coverage with free long-lasting insecticidal net (LLINs) across the 36 states and Federal Capital Territory. The new policy goal is to reach at least 80% of households with an average of two nets per household but the delivery of nets for these scaled-up distributions, supported by The Global Fund and other donors, took place over a five year period (2009-2013) on a state-by-state basis.

The net ownership figures estimated here for both states in 2010 are much lower than the current ministry target, reflecting previous policy. In order to place our state-level results in the context of previous net distribution strategy and coverage estimates, we reviewed results from the DHS 2003 [9] the study of Oresanya *et al*[36], the DHS 2008 [3], and the MIS 2010 [1] that reported zonal level estimates. In the South East zone (a group of five states including Abia, Figure 1), household net ownership of at least one net of any type was 5.8% in 2003, reportedly increased after scale up to 36.5% in 2005, decreased to 13.4% in 2008 and was up to 35% in MIS 2010. Our estimate of ownership for Abia state only in 2010 (10.1%) was surprisingly low, given that many more than 10% of households would have a vulnerable group member. It could be explained by 1) lack of net replacement since 2005, although we note that half of the nets in Abia in the present study were less than one year old; or 2) inter-state differences between Abia and other states within the South East zone, perhaps mainly reflecting the mass distribution in Anambra State that took place in 2009. Similar review of net ownership in North Central zone (a group of six states including Plateau, Figure 1), showed it to be 14.9% in 2003 [9], 19.0% in 2005 [36], 15.9% in 2008 [3] and 32.7% in MIS 2010 [1]. The latter estimate was similar to results of the current study in Plateau State only (35.1%) and suggests a large increase in net ownership from 2008 to 2010. Despite these similar estimates for state and zone, intra-zonal differences between states also likely exist in North Central zone; for example scale up to universal coverage occurred in 2009 in Niger State and likely biased the zone estimate upwards. The higher ownership overall in Plateau may be partly due to efforts by The Carter Center to increase and maintain net ownership by integrating distribution with MDA for onchocerciasis and lymphatic filariasis [10]. As in Abia, approximately half of nets observed in Plateau were less than one year old. The wide variation between states in both baseline coverage and in past and future timing of scale up distribution highlights the importance of state-level surveys in evaluating the impact of Nigeria’s mass net distribution strategy.

In both Abia and Plateau, household members had taken the initiative to purchase about half of the nets currently owned, despite differences in wealth profiles between the two states. Around one-third of nets in both Abia (37.0%) and Plateau (30.3%) had been obtained through health facilities, although not all such nets were provided free-of-charge: 9.8% and 19.4% of nets obtained from health facilities in Abia and Plateau, respectively, were reportedly purchased. One third of all nets were obtained from markets or shops, indicating significant existing demand for nets prior to statewide mass distribution, as was also observed in Enugu State, South East zone [37]. Unlike the present results, other studies of net ownership in Nigeria have observed inequity prior to mass distribution campaigns, though with conflicting trends--some report highest ITN ownership among wealthiest households [3,38], while an earlier report found inverse associations with wealth [9]. It will be important to document whether demand for nets translates into sustained net use in Nigeria once the access to free nets increases, as studies from other African countries have revealed declines in net use among households owning nets following mass distribution campaigns [39,40].

Net use estimates follow similar trends to ownership. Overall net use in 2010 estimated in this study for children under five years and pregnant women in Abia (6.0%; 5.7%, respectively) and Plateau (19.1%; 21.0%) was far below ministry target of 80% for both populations. Past trends in the South East zone for net use by children under five in all households show it was 4.4% in 2003 [9], 16.0% in 2005 [36], 14.3% in 2008 [3] and 17.4% in MIS 2010 [1]. For pregnant women in South East (not assessed in 2005) the corresponding figures were 2% in 2003, 10.2% in 2008 and 12% in MIS 2010. These indicate substantial heterogeneity in net use within the South East zone and that Abia was lower than its zone average in 2010, although this is to be expected given the low net ownership. In North Central zone, trends in net use by under fives were fairly stable at 8.9% in 2003 [9], 7.3% in 2005 [36] and 9.7% in 2008 [3] but doubled to 18.9% by MIS 2010 [1]. Pregnant women showed a similar trend at 9.2% in 2003 and 9.4% in 2008 but greater increase by MIS 2010 to 36.7%. Thus Plateau state was above average in net use by children under five (19.1%) and below average for pregnant women (21.0%) compared to its surrounding zone.

Among households that owned nets, net use by children under five and pregnant women was five-fold higher in Abia and 2.5- to 3-fold higher in Plateau compared to all households; however, only about one third (in Abia) and one half (in Plateau) of vulnerable groups reported sleeping under a net the previous night. Yet 61.3% of nets in Abia and 80.4% of nets in Plateau were reportedly used by a household member last night, indicating that nets are being used by persons other than children under five and pregnant women within households, especially in Abia. Use by other members is not surprising given that the mean number of individuals per household in each state exceeds by a factor of 3.7 the number of nets currently available per household. Analysis of the early scale-up of malaria prevention measures across sub-Saharan Africa has shown that the primary driver of net use is the relative availability of nets within households [41], and recent application of additional MERG-recommended net indicators to 2010 MIS data demonstrates that 61% and 71% of households with an ITN in South East and North Central zones, respectively, did not have enough nets for each household member (defined as one ITN per two persons) [18]. Using the same indicator, we found that 86% and 82% of households with a net (of any type) in Abia and Plateau, respectively, did not have sufficient number of nets.

Previous studies [38,41] have observed that net use among children is not significantly associated with household wealth after net distributions. In the present study, which was conducted prior to mass distributions, net use was positively associated with wealth in Plateau, but not in Abia. Interestingly, DHS 2008 and MIS 2010 both reported inverse associations between wealth and net use at the national level [1,3]. Also in contrast to other studies, which reveal a female bias in net use in Nigeria [1,42], significant differences between the sexes were not observed in either state in the present study.

Age was significantly associated with net use among all households in Abia and Plateau, which was highest in children under five and those over 20 years. This is in agreement with other surveys from Nigeria [1,38,42], which consistently observe that net usage is lowest among older children and young adults. This finding is very important given that older children are the group with highest prevalence of *Plasmodium* infection. In addition to improving access to nets, this points to a significant need for education regarding malaria prevention and net use, including by children over five years old. That this is needed even among those who own nets is illustrated by the finding that ‘not wanting to use net’ was the most common reason for nets not being hung last night and that 25% of nets in Abia had never been used.

In an effort to address these gaps, The Carter Center has developed behavior change communication (BCC) materials that emphasize strategies for increasing net use that were identified among consistent net users during focus group discussions conducted in Plateau State. In addition, BCC materials incorporate health education about lymphatic filariasis and malaria. This innovative, integrated health messaging approach was driven by the fact that both diseases share the same *Anopheles* vector and the belief that heightened awareness of LF-associated sequelae, which include swelling of the limbs (lymphedema, elephantiasis) and genital organs (hydrocele), is likely to promote increased net usage, particularly among adolescents and males.

As with any survey, there are limitations to note. Results from this study represent a single cross-sectional sample, which was collected during peak malaria season. We compared results with the 2010 MIS survey, which was conducted approximately one month after our survey. However, the DHS surveys of 2003 and 2008 were conducted during the months of March to August and June to October, respectively, which overlap periods of typically lower malaria transmission. Care should thus be taken when comparing our results with the DHS, particularly malaria parasite prevalence estimates, as well as utilization of malaria prevention measures, since net use has been observed to decline during dry seasons [43-45]. Studies of this type are also reliant upon self-reported data for many questions. In an effort to verify net ownership and ever-use of nets, survey teams visually inspected nets within households and observed whether the net was still sealed in its original packaging. However, it was not possible to verify use of net the previous night or other self-reported data. The survey was also conducted by independent groups of survey teams in each state, and unidentified sources of systematic error between teams may have biased state level estimates and the inferred differences between states. Likewise, slides from Abia and Plateau were read in separate laboratories. Although quality control was conducted by the same individual for slides from both states, systematic differences in initial slide reading between states could have occurred. Nonetheless, RDT data closely matched the overall microscopy prevalence estimates for each state, suggesting that gross errors between states, and overall, did not exist.

## Conclusions

Results from this study, which was conducted in September 2010 prior to planned mass distribution campaigns in Abia and Plateau States, document high levels of *Plasmodium* infection and anemia in both states, extremely low IRS coverage and low bed net ownership and use. Mass LLIN campaigns are expected to significantly improve access to bednets for all at-risk Nigerians and follow-up surveys are planned after distributions to evaluate progress toward ministry targets for prevention measures and impact on the disease burden of malaria.

## Abbreviations

CI: Confidence interval; DHS: Demographic and health survey; EA: Enumeration area; FCT: Federal capital territory; Hb: Hemoglobin; IPTp: Intermittent preventative therapy in pregnancy; IRS: Indoor residual spraying with insecticide; ITN: Insecticide-treated net; LLIN: Long-lasting insecticidal net; MDA: Mass drug administration; MERG: Monitoring & evaluation reference group; MICS: Multiple indicator cluster survey; MIS: Malaria indicator survey; RDT: Rapid diagnostic test; SUFI: Scale-up for impact.

## Competing interests

The authors declare that they have no competing interests.

## Authors’ contributions

AE, EE, EM, FOR, PME and PMG conceived the study. AS, AE, AEP, CO, EC, EE, JD, OO and PMG trained survey teams. AE, AEP, AS, EE, IO, JA, KA, OUO, MU and PMG conducted and supervised the survey. JN, JO, MO, PMG and SE managed survey databases. JD supervised reading of blood films and performed slide quality control, with assistance from KA. GSN, JN and PMG analyzed data. RMD produced the map. GSN drafted the manuscript. AEP, FOR and PMG contributed significant revisions to the manuscript. All authors read and approved the final manuscript.

## Pre-publication history

The pre-publication history for this paper can be accessed here:

http://www.biomedcentral.com/1471-2334/14/168/prepub
